# Predicting pandemics, predicting people: the ethical limits of AI-powered public health surveillance

**DOI:** 10.3389/fpubh.2026.1993792

**Published:** 2026-10-08

**Authors:** Maryan Ali Adam, Ayuub Axmed Osmaan, Mohamed Abdijabar Mohamed, Maryamo Maxamed Cali, Ilyas Abdullahi Khalif

**Affiliations:** 1 Faculty of Health Sciences and Tropical Medicine, Somali National University, Mogadishu, Somalia; 2 Center for Health Research and Innovation, Somali National University, Mogadishu, Somalia; 3 Benaadir Research, Consultancy and Evaluation Center, Mogadishu, Somalia

**Keywords:** AI governance, artificial intelligence, health equity, predictive epidemiology, public health surveillance

## Abstract

Artificial intelligence (AI) is transforming public health surveillance by enabling the integration of epidemiological, clinical, environmental, mobility, genomic, and digital data to detect emerging threats and anticipate disease transmission. However, the expansion from detecting outbreaks to predicting individual or group-level behaviors raises important ethical concerns related to privacy, autonomy, discrimination, transparency, accountability, and public trust. This mini-review examines the ethical limits of AI-powered public health surveillance, with particular attention to the transition from population-level epidemic prediction toward individual-level inference and profiling. It highlights how extensive data integration, algorithmic bias, unequal predictive errors, opaque models, and mission creep can undermine both equity and the legitimacy of surveillance systems. The review argues that predictive capability alone cannot establish ethical legitimacy. Instead, AI surveillance should be governed by necessity, proportionality, purpose limitation, data minimization, equity, transparency, meaningful human oversight, and mechanisms for accountability and redress. A lifecycle governance approach is further required to ensure continuous assessment of data quality, subgroup performance, model drift, unintended harms, and changing public-health needs. Ultimately, the ethical boundary of AI surveillance should be defined not by what can technically be predicted, but by what can be predicted and acted upon without compromising fundamental rights, equity, and public trust.

## 1 Introduction

Timely surveillance is foundational to outbreak control because delayed detection narrows the window for investigation, notification, and response (1). Yet conventional surveillance systems remain constrained by manual reporting, structured data dependence, diagnostic delays, limited testing early in outbreaks, and fragmented infrastructures that can miss emerging threats or detect them too late for optimal containment (2). These limitations are especially consequential for novel or zoonotic pathogens, which may circulate before laboratory confirmation or formal case definitions are established (3).

AI-powered surveillance is reshaping this landscape by expanding epidemic intelligence beyond retrospective reporting toward real-time, anticipatory inference (4). Recent work shows that machine learning, natural language processing, and related methods can integrate epidemiological, clinical, environmental, mobility, genomic, behavioral, web, and social media data to detect signals earlier and forecast where transmission risk may emerge next (5). In this sense, surveillance is no longer limited to asking what is happening; it increasingly estimates what may happen, where it may happen, and which populations may be most affected (5, 6). Open-source and syndromic signals can complement validated surveillance by identifying anomalous patterns days to weeks earlier, although they are best understood as triggers for investigation rather than substitutes for public health verification (7).

The ethical difficulty emerges because the same data integration and predictive capacity that strengthen early warning can also intensify surveillance over persons, not merely pathogens (4). As surveillance systems absorb lifestyle, mobility, search, wearable, and other non-clinical traces, they can expose sensitive attributes, enable profiling, and extend health inference into domains not originally collected for public health purposes (8). The key conceptual distinction, therefore, is between predicting population-level disease dynamics to support proportionate prevention and predicting or classifying individual behavior in ways that threaten privacy, autonomy, equity, and trust (8). This mini-review argues that AI-powered public health surveillance is not inherently unethical; its legitimacy depends on whether predictive systems remain necessary and proportionate to public health aims, protect privacy, avoid unjust discrimination, operate transparently and accountably, preserve meaningful human oversight, and sustain public trust (9). Its purpose is to examine where the boundary lies between legitimate prediction of pandemics and unjustified prediction of people.

## 2 From detection to prediction

Public health surveillance is shifting from delayed, indicator-based case counting toward predictive epidemic intelligence that analyzes heterogeneous data streams in near real time. AI systems now augment conventional surveillance by combining syndromic, digital, event-based, and laboratory signals, using machine learning, deep learning, natural language processing, anomaly detection, and time-series forecasting to identify outbreaks earlier and estimate near-term spread (9).

What changes is not only speed, but the nature of inference. Emerging systems integrate epidemiological, clinical, laboratory, genomic, environmental, climate, wastewater, mobility, and online data, and increasingly do so within multimodal or One Health frameworks (9, 10). This wider evidentiary base can improve lead time and calibration in specific contexts. For example, one open-source hybrid early-warning system integrating multiple surveillance data streams reported 83.3% sensitivity, 76.9% positive predictive value, and a median 9.3-day lead time before official confirmation in its evaluated setting (11). However, these performance metrics should not be interpreted as universally generalizable indicators of AI surveillance effectiveness. Predictive performance depends strongly on the characteristics of the pathogen, surveillance infrastructure, data completeness, population coverage, reporting practices, and local epidemiological conditions. Therefore, AI early-warning systems require context-specific validation, continuous recalibration, and assessment of local applicability before implementation in different health systems, particularly in resource-constrained settings. Yet the same integration also increases the quantity, granularity, and contextual richness of information available for inference, allowing systems to move conceptually from detection to forecasting, risk estimation, population classification, and, increasingly, individual-level inference about exposure, vulnerability, movement, or likely health status (9).

That progression matters ethically because AI can derive actionable predictions from datasets that appear ordinary in isolation. Still, current capability should not be overstated: performance depends heavily on data quality, representativeness, reporting protocols, and continuous recalibration, and real-world accuracy often falls below retrospective results, with persistent risks of false positives, false negatives, and context failure (5).

## 3 The ethical boundary

Traditional epidemiological surveillance primarily observes health events at the population level, whereas AI-enabled inferential surveillance can move beyond observation to infer hidden traits, link disparate datasets, and generate predictions from digital traces that people never knowingly supplied for public-health purposes (8). Ethical risk escalates along the pathway from observation to inference, prediction, classification, and intervention, because each step increases the power to sort people and communities rather than simply track disease patterns (8).

Privacy in this context extends well beyond medical-record confidentiality. Passive and continuous data streams such as GPS, accelerometer, text, call logs, wearables, and other ambient data enable persistent monitoring, while data linkage across sources increases both analytic reach and re-identification risk, even when data are anonymized or pseudonymized (12, 13). Secondary use further strains autonomy, especially when individuals become passive data sources with little practical control over who accesses their information, for what purpose, and for how long (8).

Consent therefore becomes structurally difficult, not merely procedurally imperfect, when data are collected indirectly, continuously, or through digital environments where opting out is infeasible (12, 14). The central ethical question is when predicting disease becomes predicting people: systems built to estimate exposure or outbreak spread can also classify mobility, health-seeking behavior, compliance, vulnerability, or other behavioral patterns, shifting epidemiological assessment toward individual or group profiling (12, 15). However, individual-level prediction is not inherently incompatible with ethical public health practice when it is narrowly defined, evidence-based, and directly linked to legitimate prevention objectives. For example, targeted vaccination strategies, exposure notification systems, and outbreak containment through contact tracing may require individual-level risk assessment to identify persons who could benefit from timely intervention or support (16, 17). In these circumstances, ethical acceptability depends not only on predictive accuracy but also on necessity, proportionality, transparency, limited data use, and the availability of appropriate safeguards against misuse (8, 14). The critical distinction is therefore between accountable individual-level risk assessment for a clearly defined public health purpose and broader forms of behavioral profiling or classification that extend beyond the original mandate of surveillance systems (14, 15).

That boundary is crossed most clearly when public-health systems are repurposed beyond necessity and proportionality. Reviews of digital surveillance repeatedly warn that outbreak tools can slide into mission creep, with overlap between public-health, commercial, border, and criminal-legal surveillance, and with historical evidence that data systems expand incrementally beyond their original mandate (18). Predictive surveillance therefore needs a defensible ethical boundary that specifies what may be predicted, at what level, from which data, for which public-health purpose, and under what human-rights-limited conditions (14, 19).

## 4 Algorithmic bias and predictive equity

Section 4 argues that the ethical boundary of AI surveillance is crossed not only when populations are watched, but when predictive systems distribute error unequally. Bias can enter at every stage of the surveillance pipeline, from data collection and integration to outcome definition, model development, validation, deployment, and interpretation (20, 21).

When data are missing, underreported, geographically sparse, or drawn mainly from digitally connected populations, models learn the health realities of the most visible groups and perform less reliably for those already underserved (22). This is especially problematic for historically marginalized communities and for low- and middle-income settings, where limited infrastructure, fragmented records, uneven connectivity, and poor local contextual fit make vulnerable populations simultaneously high risk and data poor (23, 24). Empirical work shows that missing or inequitable records can reduce predictive performance most in groups with less healthcare access, while models transferred across hospitals or countries often lose reliability when local epidemiologic and service conditions differ (25).

These distortions matter because false negatives and false positives do not fall evenly. Elevated false negatives in underrepresented groups can mean missed outbreaks, delayed intervention, or exclusion from preventive resources, whereas false positives can trigger unnecessary surveillance or misdirect scarce capacity (26). A model can therefore show good overall accuracy while remaining poorly calibrated for racialized, low-income, or otherwise marginalized subgroups (27). For AI public-health surveillance, equity is thus a core performance criterion, not an optional ethical add-on (26).

## 5 Black-box explainability and accountability in public health AI

Following the bias and equity concerns in Section 4, the ethical risk becomes concrete when unequal or incorrect predictions are used in decisions that neither professionals nor communities can meaningfully understand or challenge.

Algorithmic opacity matters in public-health surveillance because high-performing models often function as black boxes, creating a tension between predictive accuracy and interpretability precisely where decisions must remain publicly justifiable and democratically accountable (28). Technical explainability helps reveal variables, pathways, or output logic, but explanation alone does not solve the deeper problem of epistemic opacity, nor does it guarantee that affected communities or frontline practitioners can evaluate or contest a prediction in meaningful terms (29). In practice, accountability is often diffused across developers, vendors, data managers, epidemiologists, public-health authorities, and end users, while responsibility after harm can collapse unfairly onto the human operator, producing a liability sink or “moral crumple zone” (30).

Human oversight is therefore ethically meaningful only when it involves real capacity to question, override, and interrupt algorithmic action, rather than merely approving recommendations under time pressure or institutional deference (31). Responsible surveillance also requires explicit communication of uncertainty, intended use, subgroup limits, data limitations, and model drift, because static accuracy metrics can conceal evolving failure modes after deployment (4). Accountable AI surveillance, then, depends on clearly assigned responsibility, meaningful human oversight, transparent uncertainty, and mechanisms for review, correction, and redress when predictions cause harm. Nevertheless, explainability requirements should be considered proportionately to the context and potential consequences of AI deployment. In some public-health applications, highly complex models may provide valuable predictive performance even when complete technical interpretability is difficult to achieve. However, opacity becomes ethically problematic when predictions directly influence decisions affecting individuals, communities, or access to health resources without adequate mechanisms for validation, accountability, and human review (29). Therefore, the objective should not necessarily be complete transparency of every computational process, but meaningful transparency regarding model purpose, data sources, limitations, uncertainty, performance across population groups, and the basis for human decision-making (29, 32). Similarly, meaningful human oversight requires more than the presence of a human decision-maker; it requires institutional capacity, trained personnel, clear accountability structures, authority to challenge algorithmic outputs, and procedures for correction and appeal (31, 33). In fragile and resource-constrained settings, effective oversight may require balanced collaboration between international technical expertise and local public-health institutions to ensure that AI systems are scientifically robust, contextually appropriate, and aligned with community priorities (32, 33). International frameworks, including the European Union Artificial Intelligence Act and emerging global health AI governance guidance, increasingly emphasize risk-based regulation, human oversight, transparency, and accountability, although further adaptation may be required to address implementation realities in low-resource health systems (31, 32).

## 6 Public trust and predictive surveillance

Technically accurate AI surveillance can still fail if affected populations experience it as intrusive, opaque, or discriminatory, because public health legitimacy depends on social acceptance as much as analytic performance. Trust is consistently described as a social foundation for implementation, and evidence across public health AI, digital epidemiology, and surveillance ethics shows that performance alone is insufficient without procedural fairness, transparency about data use, and meaningful avenues to question decisions (16).

This is why earlier concerns about privacy violations, bias, and opaque decision-making become operational problems, not merely ethical ones. When people do not know what data are collected, how they are processed, or why they are reused, they cannot assess participation and are more likely to perceive surveillance as illegitimate (8). Continuous monitoring and unclear governance can then generate fear, distrust, and disengagement, while epidemic conditions themselves make populations more vulnerable to rumors, conspiracies, and misinformation (34).

Communities therefore cannot be treated simply as data sources; they must be recognized as stakeholders in how predictive surveillance is designed, communicated, and overseen. Studies on data justice, citizen governance, and responsible surveillance argue for community oversight, advisory panels, accessible communication, and channels for feedback, complaint, and redress so that trust is co-produced rather than presumed (15). Trust is not a messaging problem alone. It is the cumulative judgment that surveillance is fair, proportionate, accountable, and respectful of rights; for that reason, public trust should be treated as an operational requirement for sustainable epidemic intelligence (35).

## 7 A rights-preserving model for AI surveillance

AI-powered public-health surveillance can be ethically justified, but only under a rights-preserving model that treats predictive capacity as subordinate to public health necessity, human rights, and accountable governance. Across the literature, ethical legitimacy turns not on whether AI is powerful, but on whether its use is necessary for a defined health threat, proportionate to the risk, limited to a public-health purpose, restrained in data collection, and governed by meaningful human responsibility (16, 32).

The first principle is necessity. AI surveillance should be used only when a specific public-health objective cannot be achieved adequately through less data-intensive or less intrusive means. Public health ethics guidance consistently frames permissibility, effectiveness, proportionality, accountability, and public participation as threshold conditions for deployment rather than after-the-fact justifications (17). This makes problem definition morally prior to technical capability: agencies should first identify the threat, the decision that must be supported, and the evidence that AI materially improves response capacity over conventional surveillance (16).

The second principle is proportionality. Even when surveillance serves a legitimate aim, its intrusiveness must be justified by the magnitude and probability of harm, with special attention to civil liberties, privacy, and the unequal burdens borne by marginalized groups (15, 18). A proportional system therefore rejects blanket monitoring where targeted, time-limited, or aggregated approaches would suffice, and it must remain sensitive to digital divides and structural inequities that can make efficiency gains ethically regressive (36).

The third and fourth principles are purpose limitation and data minimization. Surveillance systems require clearly specified public-health purposes, explicit rules against secondary use, and safeguards against the function creep repeatedly identified in digital and AI health surveillance (8). Predictive utility does not justify collecting every technically available stream of behavioral, locational, or social data; responsible governance instead limits collection to data necessary for the stated function, pairs access controls with privacy-preserving methods, and reviews utility-protection trade-offs case by case (16).

The fifth principle is human accountability. Because opacity, stakeholder fragmentation, and automation can diffuse responsibility, consequential decisions must remain contestable, reviewable, and attributable to identifiable institutions and decision-makers (37). Transparency, equity, and community participation are not separate add-ons but conditions that make accountability real: affected populations need understandable information, avenues for redress, and meaningful roles in design and oversight (8).

This framework implies an ethical ceiling for predictive surveillance. When systems move from population-level risk detection toward persistent individual profiling, behavioral inference, opaque scoring, or automated intervention, the public-health justification can become insufficient even if predictive performance improves (8, 37). AI surveillance is therefore ethical only when it remains necessary, proportionate, purpose-bound, data-minimizing, equitable, transparent, and answerable to human judgment.

## 8 Governance framework for ethical AI surveillance

A practical governance framework for AI-powered public-health surveillance should treat oversight as a lifecycle function, not a one-time approval step. Recent governance literature consistently argues that trustworthy health AI requires processes that begin with problem definition and continue through validation, deployment, monitoring, updating, and, when justified, retirement (32, 33).

The pathway should begin with a clearly specified public-health need. Governance frameworks increasingly place need and problem identification at the start because institutions must first establish that AI addresses a genuine surveillance problem, clarify intended users and decisions, and assess whether AI is appropriate relative to less intrusive alternatives (33). This is where ethical impact assessment should test necessity and proportionality: what data are needed, for what purpose, under what legal authority, and with what foreseeable risks if predictions are wrong or repurposed (38, 39).

Next, data governance and bias assessment should determine whether the system is fit for purpose before it is ever used operationally. Across the literature, robust pre-deployment review includes representative data, traceable data provenance, privacy and security protections, scrutiny of proxy variables, subgroup analysis, calibration, and local validation under actual decision thresholds rather than headline accuracy alone (16, 26). Equity must be assessed as a design property, because underrepresentation and poorly chosen proxies can systematically encode structural disadvantage into surveillance outputs (26).

Before and during deployment, community oversight and human decision-making remain essential. Public-health AI governance increasingly emphasizes independent review bodies, community advisory mechanisms, transparent documentation, and public communication so that surveillance practices remain aligned with stakeholder values rather than institutional convenience alone (8, 38). Deployment should preserve human authority, require user training, communicate uncertainty, and create clear override and correction pathways for harmful or implausible outputs (38, 40).

After go-live, continuous auditing is indispensable because both models and epidemiological conditions change. Post-deployment governance should monitor drift, calibration, incident reports, feedback loops, subgroup performance, workflow effects, and unexpected harms, with predefined thresholds for retraining, suspension, rollback, external review, or replacement (41, 42). This adaptive approach is especially important in public health, where care-seeking patterns, data streams, policy priorities, and disease threats evolve rapidly (16).

For that reason, predictive surveillance systems should not become permanent infrastructure by default. Several recent frameworks explicitly argue for reassessment, decommissioning, or retirement when systems are no longer accurate, equitable, legally defensible, or operationally useful, and for guarding against function creep as surveillance capacities expand beyond their original mandate (16, 43). Trustworthy predictive surveillance therefore depends not only on technical performance, but on institutional capacity, regulatory oversight, continuous monitoring, ethical review, and meaningful public participation across the full AI lifecycle (16, 44). Fragile and resource-constrained health systems demonstrate why context-specific governance is essential for responsible AI-powered surveillance. Somalia provides an important example, where recurrent infectious disease threats, humanitarian pressures, limited surveillance capacity, and uneven health-system infrastructure create both opportunities and ethical challenges for AI adoption (45, 46). In such settings, predictive surveillance may strengthen early warning and outbreak response, but implementation requires careful consideration of data quality, representativeness, institutional readiness, and local ownership (5). Limited population coverage and incomplete health information systems may increase risks of algorithmic bias, while externally developed models may not adequately reflect local epidemiological patterns, healthcare access conditions, or community priorities (5, 23). Therefore, AI surveillance in fragile settings should be approached not only as a technological innovation but as a governance challenge requiring locally adapted frameworks, national institutional leadership, transparent partnerships, and meaningful engagement with affected communities (33).

## 9 Conclusion

AI-powered surveillance offers substantial potential to strengthen epidemic intelligence by enabling earlier detection, prediction, and response to emerging health threats. However, the expansion of surveillance from predicting population-level disease dynamics to predicting individual behavior, vulnerability, or health status creates significant ethical risks. Privacy intrusion, algorithmic bias, unequal errors, opacity, mission creep, and declining public trust can undermine the very public-health objectives these technologies are intended to support. The central challenge is therefore not whether AI should be used in surveillance, but where its legitimate boundaries should be drawn. AI surveillance should remain necessary, proportionate, purpose-limited, data-minimizing, equitable, transparent, and subject to meaningful human accountability. Governance must also extend throughout the AI lifecycle, from problem definition and data assessment to deployment, continuous auditing, and eventual decommissioning when systems no longer meet ethical or public-health requirements. Predicting pandemics can strengthen health security; predicting people without adequate safeguards can weaken it. The future of public-health AI should therefore be defined not by the maximum amount of information that can be collected or inferred, but by the ability to use predictive intelligence while preserving rights, equity, accountability, and public trust.
